# mSphere of Influence: Peering through a Keyhole into the Unseen World

**DOI:** 10.1128/mSphere.00980-19

**Published:** 2020-01-29

**Authors:** Sarah L. Lebeis

**Affiliations:** a Department of Microbiology, University of Tennessee, Knoxville, Tennessee, USA

**Keywords:** culture collection, genome sequencing, microbiomes

## Abstract

Sarah Lebeis studies the assembly and function of plant microbiomes. In this mSphere of Influence article, she reflects on how the paper “Functional Overlap of the *Arabidopsis* Leaf and Root Microbiota” (Y. Bai, D. B. Müller, G. Srinivas, R. Garrido-Oter, et al., Nature 528:364-369, 2015, https://doi.org/10.1038/nature16192) provided a roadmap for how large culture collections composed of well-characterized bacterial isolates provide essential resources to test hypotheses concerning microbial communities.

## Abstract (precis)

Sarah Lebeis studies the assembly and function of plant microbiomes. In this mSphere of Influence article, she reflects on how the paper “Functional Overlap of the *Arabidopsis* Leaf and Root Microbiota” (Y. Bai, D. B. Müller, G. Srinivas, R.

## COMMENTARY

In “Functional Overlap of the *Arabidopsis* Leaf and Root Microbiota,” Bai et al. (1) reveal microbial activities associated with plant colonization of different plant organs by establishing a culture collection of 400 bacterial isolates generated from Arabidopsis thaliana leaves and roots. This collection, which represents the majority of taxa identified by culture-independent 16S rRNA gene amplicon sequencing, was developed into a unique scientific community resource when the cultures were deposited into a special collection in Germany’s DSMZ and their genomes were sequenced. Bai et al. also uncovered inherent differences between leaf-isolated bacterial strains and those isolated from roots or soil by establishing synthetic community inocula that assemble robust community structures with distinguishable functional potential. The combination of comparative genomic and competitive colonization studies demonstrated that although strains may be assigned to similar taxa by their 16S rRNA gene sequences, they possess unique attributes that influence their colonization of different plant tissues. This work highlights the importance of testing hypotheses generated by culture-independent approaches and provides its community with resources for far-reaching future research.

The advent of amplicon sequencing allowed researchers an incredible view into their study system of choice, but these types of data provide the context and potential players, not necessarily the answers to their scientific questions. The research question here was whether there are similarities and differences in the functional potential of bacteria that colonize above- and belowground plant tissues. Bai et al. describes the impressive efforts by two collaborating groups to culture diverse bacterial strains from leaves, roots, and soil. They quantify how well their culture collection recreates each microbiome and carefully determine the predicted functional diversity present in their culture collection. Finally, when strains isolated from either above- or belowground sources were competed against each other on reciprocal tissues, inherent differences between these microbes were revealed. These studies provide a crucial reminder that although 16S rRNA gene amplicon sequencing studies transformed the way that each of us thought about the microbial composition of our scientific study host and environment systems, 16S rRNA is a single gene that does not reveal the diversity of the potential activities of those microbes.

When I first started working with amplicon sequencing data sets, facing a read abundance table full of unfamiliar microbes, the idea of trying to make biological sense from these unknown entities seemed overwhelming. Our early attempts to simply isolate as many members of the *Arabidopsis* microbiome as possible initially felt like grasping at straws while drowning in a sea of data. We had no idea how well these exemplars captured the breadth of actively participating microbiome members. Bai et al. demonstrated that it was feasible to uncover important, but previously untested, biology that shapes microbial communities and influences host health through culturing efforts. Forming synthetic community modules from a large, well-characterized microbe collection can be used to address hypotheses generated from culture-independent sequencing approaches, which allows scientists to reveal the overlapping mechanisms of community assembly, along with both the potential and realized community functions. While we know that we do not capture all active members of these microbial communities, culture collections and the synthetic communities derived from them are beginning to make complex systems tractable and predictably mutable to tackle many research questions.
